# Cooling via one hand improves physical performance in heat-sensitive individuals with Multiple Sclerosis: A preliminary study

**DOI:** 10.1186/1471-2377-8-14

**Published:** 2008-05-12

**Authors:** Dennis A Grahn, Julie vLS Murray, H Craig Heller

**Affiliations:** 1 Department of Biological Sciences, Stanford University, Stanford, CA 94305, U.S.A.

## Abstract

**Background:**

Many individuals afflicted with multiple sclerosis (MS) experience a transient worsening of symptoms when body temperature increases due to ambient conditions or physical activity. Resulting symptom exacerbations can limit performance. We hypothesized that extraction of heat from the body through the subcutaneous retia venosa that underlie the palmar surfaces of the hands would reduce exercise-related heat stress and thereby increase the physical performance capacity of heat-sensitive individuals with MS.

**Methods:**

Ten ambulatory MS patients completed one or more randomized paired trials of walking on a treadmill in a temperate environment with and without cooling. Stop criteria were symptom exacerbation and subjective fatigue. The cooling treatment entailed inserting one hand into a rigid chamber through an elastic sleeve that formed an airtight seal around the wrist. A small vacuum pump created a -40 mm Hg subatmospheric pressure enviinside the chamber where the palmar surface of the hand rested on a metal surface maintained at 18–22°C. During the treatment trials, the device was suspended from above the treadmill on a bungee cord so the subjects could comfortably keep a hand in the device without having to bear its weight while walking on the treadmill.

**Results:**

When the trials were grouped by treatment only, cooling treatment increased exercise durations by 33% (43.6 ± 17.1 min with treatment vs. 32.8 ± 10.9 min. without treatment, mean ± SD, p < 5.0·10^-6^, paired t-test, n = 26). When the average values were calculated for the subjects who performed multiple trials before the treatment group results were compared, cooling treatment increased exercise duration by 35% (42.8 ± 16.4 min with treatment vs. 31.7 ± 9.8 min. without treatment, mean ± SD, p < 0.003, paired t-test, n = 10).

**Conclusion:**

These preliminary results suggest that utilization of the heat transfer capacity of the non-hairy skin surfaces can enable temperature-sensitive individuals with MS to extend participation in day-to-day physical activities despite thermally stressful conditions. However, systematic longitudinal studies in larger cohorts of MS patients with specific deficits and levels of disability conducted under a variety of test conditions are needed to confirm these preliminary findings.

## Background

For individuals with multiple sclerosis (MS), heat-induced symptom exacerbation can be a limiting factor for physical activity. Up to 80% of individuals with MS report symptom exacerbation associated with heat stress resulting from exercise, exposure to elevated environmental temperatures, or both [1-5]. MS symptoms vary between individuals and often include deficits associated with coordinated movement such as: muscle weakness, muscle spasms, ataxia, and visual problems. Heat- or exercise-induced symptom exacerbations are a transient expression of new symptoms, or a worsening of existing symptoms. Exercise-induced MS symptom exacerbations are the result of an accumulation of metabolic heat generated by the working skeletal muscles [6-8]. To mitigate the worsening of symptoms during exercise, heat production must be reduced or heat loss increased. Heat production is proportional to work output and, thus, can only be reduced by decreasing the level of physical activity. Currently available means for increasing heat loss are either: 1) selecting an optimal (i.e., cool) environment or 2) creating a cool microclimate by wearing cooling garments. A means to directly facilitate heat removal from the body core could forestall the onset of symptom exacerbation and enable heat-sensitive individuals to increase their endurance capacity during physical activity or exposure to elevated environmental temperatures.

Specialized subcutaneous vascular structures [arteriovenous anastomoses (AVAs) and associated retia venosa] underlie the non-hairy skin surfaces of the human body (the palms of the hands, the soles of the feet, the ears, and face) [9]. These unique vascular structures distribute a large volume of blood over a two dimensional plane just beneath the skin surface. During extreme heat stress, blood flow through the subcutaneous AVAs can be as high as 8 l/min (60% of the cardiac output) [10]. Blood flow through these specialized vascular structures provides a means for heat accumulated within the body to be transferred efficiently to the external environment [11]. The venous blood returning from these vascular beds can have a significant effect on the temperature of the body core, and especially on organs and active muscles that are receiving high percentages of the cardiac output [12-18].

We have developed a portable means for optimizing heat exchange through the retia venosa underlying the non-hairy skin surfaces of the hands and feet [19-21]. A hand or foot is enclosed in an air tight chamber and a pressure differential is used to expand the rete venosum of the enclosed appendage and thereby draw an increased blood volume into it. Through the combined local application of low level subatmospheric pressure and an appropriate thermal load, it is possible to transfer a substantial amount of heat into or out of the circulating blood and, thereby, directly manipulate core temperature using only a single hand [19-22]. This heat extraction method decreased the rates of core temperature rise and improved the physical performances of fit and active individuals during aerobic exercise in a hot environment [20,22]. We questioned whether use of this technique would provide a similar benefit to heat-sensitive individuals with MS.

## Methods

### Subjects

Inclusion/exclusion criteria for this study were a diagnosis of MS, a history of heat sensitivity, engagement in a regular exercise/physical fitness program, and the ability to ambulate independently. Six males and six females volunteered to participate in the study. The subjects had mid-range Kurtzke Expanded Disability Status Scale (EDSS) scores ranging from 3.5 (fully ambulatory but with moderate disability in 1 functional system and mild disability in 1 or 2 functional systems) to 6.0 [intermittent or unilateral constant assistance (cane, crutch or brace) required to walk 100 meters with or without resting]. The sex, age, MS classification, and primary symptoms of each subject are tabulated in Table 1. Informed consent was obtained from each subject using an instrument approved by the Stanford University Institutional Review Board. Each subject was assigned an alphanumeric identifier which was used thereafter in accordance with Health Insurance Portability and Accountability Act guidelines.

**Table 1:** Gender, age, and diagnosis of individual subjects.

Subject	Gender	Age	Type	Primary symptoms
1	M	45	RR	Leg muscle weakness, cognitive difficulties
2	M	63	RR	Spasticity, cognitive difficulties
3	M	42	RR	Fatigue, balance, cognitive difficulties
4	F	53	RR	Fatigue, balance, spasticity
5	M	46	RR	Leg motor control, balance
6	M	55	SP	Limb weakness
7	F	55	RR	Fatigue, postural muscle weakness
8	F	55	SP	Leg motor control
9	M	53	RR	Ataxic gait, cognitive difficulties
10	F	56	RR	Leg muscle weakness, spasticity, fatigue
11	F	42	RR	Vertigo^a^
12	F	45	RR	Limb weakness^a^

RR-Relapsing Remitting, SP- Secondary Progressive.

^a ^subject withdrew from study due to self-reported relapse.

### Facilities and Monitoring equipment

Trials were conducted on a stationary treadmill (Nordic Track model 9800). The ambient conditions were 23.0 ± 1.0°C, 10–25% relative humidity. Heart rate monitors/data loggers (model S810, Polar Electro Oy, Kempele, Finland) collected heart rate data at 5 second intervals. Hand-written data logs noted subject identifier, date, treatment, exercise duration, and miscellaneous comments. Exercise durations were timed using a commercially available stop watch (four-channel alarm timer, VWR).

### Heat Extraction Device

The heat extraction device (AVAcore Technologies, Ann Arbor, MI) consisted of a rigid chamber into which one hand was inserted through an elastic sleeve that formed a flexible airtight seal around the wrist. The rigid chamber was connected to a pressure sensor and a vacuum pump. The vacuum pump created a slight subatmospheric pressure environment inside the chamber (-40 mm Hg) where the palm rested on a curved metal surface that was maintained at 18–22°C by temperature-controlled water circulated beneath the metal surface. The device was suspended from the ceiling by an elastic cord during the exercises to reduce the impact of wearing the device on gait, stride, and posture while walking.

### Protocol

Each subject was equipped with a heart rate monitor prior to all trials. The stop criterion for all exercise trials was symptom exacerbation which included subjective fatigue and gait or posture deterioration. Baseline assessments of individual physical performance capacities were conducted on day 1. For this assessment the subject began walking on a level treadmill at 0.8 Km/hr (0.5 mi/hr). At three minute intervals the speed or slope of the treadmill was alternately increased. The speed was increased by 0.8 Km/hr increments up to a maximum speed of 5.6 Km/hr (3.5 mi/hr). The slope of the treadmill was increased by 1% increments. Once the treadmill speed reached 5.6 Km/hr, only the slope was incrementally increased. For the subsequent experimental trials the speeds and slopes of the treadmill were adjusted to approximate 65% of the maximum work load achieved by the individual subject during his or her baseline trial. The workload for each subject was kept constant for the pair of experimental trials.

An experimental trial consisted of the subject walking on the treadmill at a predetermined speed and slope until a stop criterion was reached. Experimental trials were initiated not less than two days after the baseline assessment trial and separated by a minimum of two days and a maximum of seven days. The subjects returned to the laboratory at the same time of day for all of their trials. All subjects performed a minimum of two experimental trials, one without using the heat extraction device and one using the device. The daily experimental routine consisted of a fifteen min rest in a sitting position, a 3 min warm-up walk at 1.6 Km/min and 0% slope, the experimental trial, and 30 min of seated recovery. On days when cooling treatments were administered, the heat extraction device was donned prior to the onset of treadmill activity. The order of the treatments was randomized. A flip of a coin before the first of two paired trials determined treatment order. Water was available to the subjects *ad libitum *throughout the trials.

### Statistical Analysis

Heart rates were plotted for each trial and exercise durations tabulated on a spreadsheet (Microsoft Office Excel). The exercise duration data were analyzed in two ways: 1) If subjects completed more than one set of paired trials, mean exercise durations were calculated for each individual's control and treatment trials. The individual subjects' mean exercise duration data were then grouped according to treatment. Group mean and standard deviation were calculated for each treatment and the data were compared with a paired t-test. The proportional effects of treatment were determined by dividing exercise duration with heat extraction by exercise duration without heat extraction. 2) All sets of paired trial data were grouped according to treatment and the data plotted as exercise duration with treatment vs. exercise duration without treatment. The plotted data were subjected to a curve fitting analysis using the data analysis tools provided with Microsoft Office Excel. Trendlines (based on linear, log, polynomial, power and exponential equations) were calculated for the graphed data and the variance in the data accounted for by each function calculated. The variances were then compared to determine best fit curves. Mean and standard deviations were calculated for treatment grouped data and the data were compared with a paired t-test.

## Results

A total of 88 experimental trials were conducted on the twelve subjects. Two subjects experienced relapses and dropped out of the study without completing a set of paired trials. Twenty six data sets that met the criteria for paired trials (identical work loads and a maximum of seven days separating the trials) were collected from the remaining ten subjects. Cooling treatment increased performance (Table 2). When the individual subject's mean data were analyzed according to subject and treatment, treatment extended exercise duration by an average of 35% (42.8 ± 16.4 with cooling compared to 31.7 ± 9.8 minutes without cooling, mean ± standard deviation, n = 10, p < 0.003, paired t-test) and ranged from 8% to 65%.

**Table 2:** Treadmill speed and slope, number of paired trials, mean trial times and effect ratio (exercise duration with cooling/exercise duration without cooling): individual subjects and grouped data

Subject	Speed^a ^(Km/h)	Slope^a ^(%)	Number of paired trials	Exercise duration (min)		Cooling effect (ratio)
					
				Control	Cooling	
1	4.8	5 – 6	3	17.2	22.9	1.34
2	0.8	0	1	20.0	32.0	1.60
3	4.0	5 – 6	3	20.7	22.1	1.07
4	1.3	0	1	25.0	28.3	1.13
5	3.2 – 4.8	6	4	36.4	44.8	1.23
6	4.8	7 – 8.5	3	37.4	49.8	1.33
7	4.0	6 – 7	5	38.4	51.6	1.34
8	2.4	0	2	39.8	43.2	1.09
9	3.2	0	2	39.9	67.5	1.69
10	3.2	5.5 – 6	2	42.3	65.8	1.55

Group data ^b^						

Mean ± Standard Deviation				31.7 ± 9.8	42.8 ± 16.4	1.35 ± 0.22

^a ^Slopes and speeds of the treadmill were adjusted between sets of paired trials.

^b ^P < 0.01, paired t-test

**Figure 1:**
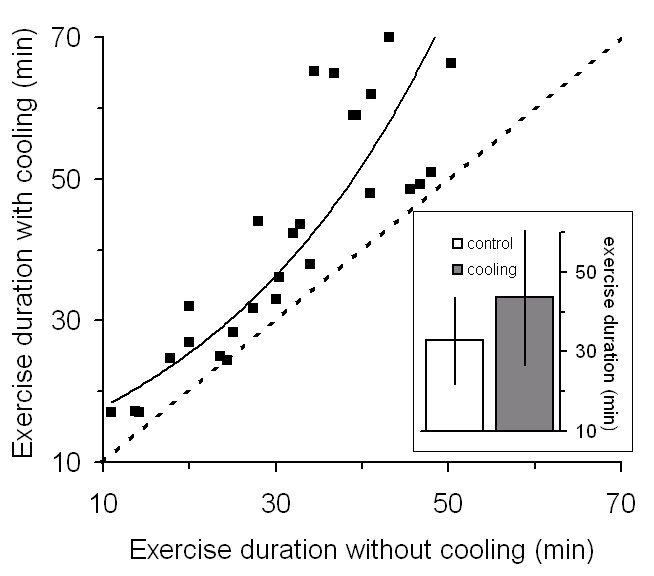
**The effect of hand cooling on exercise duration: a comparison of twenty-six paired treatment trials.** The effect of treatment (cooling) was affected by exercise duration during the control condition. The longer the duration of exercise in the control condition, the greater the cooling treatment effect. An exponential function (y = 12.505e^0.0356x^) accounted for 90% of the variance in the data. The solid line was generated by the exponential function; the dashed line represents unity. Inset: mean ± SD exercise duration with treatment and without treatment (43.6 ± 17.09 min. with treatment vs. 32.8 ± 10.9 min. without treatment (p < 5.0·10^-6^, two tailed paired t-test, n = 26).

When the exercise duration data were analyzed according treatment only (n = 26), mean exercise duration with treatment was 43.6 ± 17.09 min (mean ± SD) compared to 32.8 ± 10.9 without treatment: a mean increase of 33% (p < 5.0·10^-6^, two tailed paired t-test). The magnitude of the treatment effect was correlated with exercise duration during the control trials (Figure 1). Ninety percent of the variance in the improvements with the treatment could be accounted for by an exponential function fitted to the data (y = 12.505e^0.0356x ^with y and x being exercise duration with treatment and without treatment, respectively).

**Figure 2:**
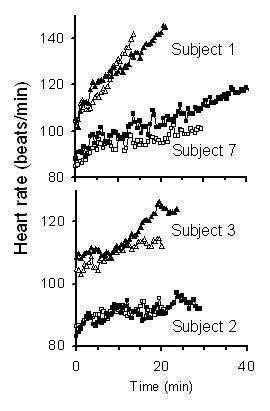
**The effect of cooling on heart rate during exercise.** Examples of heart rates during paired control and experimental trials from 4 subjects. Open symbols control, closed symbols cooling. While treatment (cooling) had little effect on heart rate, there were substantial differences between individual subject heart rates. This inter subject variability in heart rate was due in part to differences in work loads among subjects.

There were no discernable patterns in heart rates (Figure 2). Cardiac drift – a rise in heart rate during sustained fixed load exercise – was observed in all trials. Initial heart rate, final heart rate, and rate of cardiac drift (change in heart rate/time) varied among the subjects and trials. There was no significant difference between treatment groups in initial heart rates [87 ± 7 beats per minute (bpm) with cooling compared to vs. 88 ± 10 bpm without cooling, mean ± standard deviation, n = 10, p ≤ 0.56, paired t-test]. Treatment significantly affected maximum heart rates (123 ± 18 bpm with cooling compared to 118 ± 20 bpm without cooling, mean ± standard deviation, n = 10, p ≤ 0.03, paired t-test). The higher maximum heart rates observed during the cooling treatment trials were likely related to increased exercise durations with the cooling treatment. However, the rates of cardiac drift were proportional to neither work loads nor exercise duration times. Treatment did not affect the cardiac drift in a consistent manner: in some cases treatment increased cardiac drift; in others, treatment decreased cardiac drift; while, in others, treatment had no effect heart rate patterns.

Anecdotally, most subjects reported feeling better during the cooling trials. Several subjects reported that instead of experiencing the usual progressive fatigue when exercising, their symptoms occurred in waves when they used the heat extraction device. Another subject reported experiencing an unusual set of symptoms when treated. Instead of feeling "cloudy" during exercise, he felt a tingling in his legs.

## Discussion

Under the experimental conditions described in this report, the use of a device that facilitates removal of heat from the circulating blood provided a performance benefit to individuals with MS who had a history of transient worsening of symptoms associated with conditions that would impose a heat load on their bodies. In this study, the dependent variable was physical performance while the independent variable was the application of a cooling technique. Methodological limitations in the study design may have weakened the association between the independent and dependent variables and should be considered when evaluating the merit of the data presented in this report. We are aware of three methodological limitations in the study design: 1) the lack of a placebo control, 2) the researchers were not blinded to the treatments, and 3) the absence of a temperature measure or a subjective assessment of thermal comfort.

To facilitate heat transfer into or out of a body, it is necessary to apply a heat source or sink to a surface of the body. The sensations of temperature result from the differential activation of warm and cold cutaneous temperature sensors that respond to changes in local skin temperature and, thus, individuals can perceive the presence of a thermal source or sink when it is applied to the body surface [23]. Blocking the afferent input from the cutaneous thermosensors in local skin regions would be a way to eliminate the sensory input, but use of a nerve block to control for a placebo effect seemed excessive for this preliminary study. We are unaware of a practical means to blind subjects to treatment when a treatment entails applying a thermal stimulus to the skin surface.

In these studies the researchers as well as the subjects were aware of the treatments being applied during a given trial. A common confound in exercise performance trials is researcher bias. In trials on healthy subjects with subjective stop criteria, peak performance can be influenced by external motivational factors such as differences in the researcher's confidence to push the subject. Researcher bias, while always a potential confound, likely had little influence on exercise duration in these trials on individuals with MS because the primary stop criterion for exercise was an exacerbation of physical symptoms rather than being related to motivational factors. Researcher bias could have been eliminated from these trials by physically isolating the subjects from the researcher. However, since these subjects were physically compromised and exercising on a treadmill often to near the point of physical collapse, it was deemed more prudent to have the researchers directly observe the subjects during the trials and be immediately available to assist the subjects at the termination of exercise.

Neither a real measure of body temperature nor a subjective assessment of perceived temperature was monitored during these trials. The effects of temperature on individuals with MS are well known: increases in temperatures often exacerbate symptoms while decreases in temperatures can improve them [4,24-26]. However, the magnitude of thermal stimulus necessary to elicit a symptom exacerbation in MS patients can be minor and may be insufficient to be noted as a change in a measured deep body temperature [27]. In previous studies substantial improvements in fatigue, muscle strength and standing balance were observed when individuals with MS were actively cooled using cooling garments, but there was not an associated effect of lowering tympanic temperatures [28]. Since the effects of cooling have been demonstrated without an associated effect on core temperatures and placement of a core temperature probe can be stressful and unpleasant for the subjects, core temperatures were not measured in this study. The cause of the physical performance improvements could be multi-factorial and thus, without a direct measure of core temperature, we can only speculate that the treatment used in these studies provided a cooling benefit which resulted in the performance improvement. Given the multitude of published reports on the effects of thermal manipulation of the hands or feet on core temperatures and performance [12-22] and on the effects of thermal manipulations on performance [8,27-29] and symptom exacerbation [24-26] in individuals with MS, this is a reasonable inference.

The effects of manipulating the internal thermal condition of MS patients by delivering a thermal load to a single appendage have been noted previously [24,25]. In 1950 Guthrie observed that MS patients became extremely weak while sitting in a hot water bath. A similar response could be elicited in those subjects by simply immersing a single arm or leg in hot water. An arterial tourniquet during arm immersion suppressed the symptom exacerbations. At the time it was theorized that the underlying mechanism for the heat response of the MS patients to limb immersion was due to a humoral factor, a reflex vasomotor response, or to a change in cerebral blood flow. An alternative explanation put forth at that time and discounted was that adequate amounts of heat were being delivered through one appendage to increase core temperature thereby inducing symptom exacerbation.

MS is a disease of the central nervous system (CNS). Presumably, it is increases in CNS temperature, rather than body temperature per se, that cause heat related symptom exacerbations [27]. The brain and spinal cord are metabolically stable tissues that generate metabolic heat at a relatively constant rate [30]. Heat is removed from CNS tissues through the vascular system. Thus, the temperature of the CNS is determined primarily by the temperature of the arterial blood [30,31]. If the temperature of the arterial blood is elevated, CNS temperatures will rise. Heat is produced by working skeletal muscles during physical activity. Heat generated by skeletal muscles is removed from the muscle by the circulation and the warmed venous blood returning from active skeletal muscles is mixed with other venous return blood as it passes through the heart and pulmonary circulation. Any thermal manipulation that delivers a cool thermal load to the venous return blood will counteract the effects of the heat produced by active skeletal muscles. By accessing the circulating blood flowing through the retia venosa underlying the non-hairy skin surfaces it is possible to directly cool substantial volumes of venous blood.

The results reported here agree with those of previous studies that examined the effects of cooling on ambulatory performance in heat-sensitive individuals with MS; cooling can improve performance [8,29]. In the previously reported studies the subjects were cooled for 30 min prior to fixed-duration exercise bouts followed by evaluation[8] or for 60 min between evaluations [29]. Evaluation of physical performance was time and number of steps required to complete a 25 foot walk. In those studies the thermal loads were delivered to the general skin surface via a circulating water garment[29] or by cold water immersion [8]. Applying a cool load to the general skin surface directly affects the thermal inertia of the skin and underlying tissues. However, application of a thermal load to the general skin surface has little direct effect on the metabolically active core organs because the hairy skin surfaces are poorly perfused and therefore insulate the body core from the external thermal environment [32,33]. Applying cooling to the general body surface takes advantage of the thermal inertia of poorly perfused superficial and peripheral tissues, however, to store a cold load [8]. This cold load in the periphery then passively absorbs heat conducted into it by the blood flow and is the reason that precooling can improve endurance [34]. Conversely, with heat exchange involving the retia venosa, the thermal load is applied directly to the blood flowing through the palm during exercise. Cooling a volume of circulating blood directly affects the highly perfused core organs.

Precooling has been reported to result in lower heart rates during subsequent fixed load exercise in MS subjects [8]. The difference in effect of cooling treatment on heart rate between the previous report and the results reported here is likely methodological. Precooling the peripheral tissues likely induced a vasoconstriction response that reduced blood flow to the skin and peripheral tissues. Peripheral vasoconstriction increases peripheral resistance resulting in a rise of arterial pressure and a subsequent baroreceptor response decreasing heart rate [35]. In the present study the temperature of the water perfusing the cooling device was maintained at 18–22°C to avoid triggering local vasoconstriction which would, in addition to increasing vascular resistance, reduce blood flow through the heat exchange vascular structures and, thereby, decrease heat transfer.

The ability to effectively remove heat from the body could substantially improve the daily lives of heat-sensitive individuals with MS. Critical body surface regions for direct heat exchange are the non-hairy regions that contain the subcutaneous arteriovenous anastomoses and retia venosa. Heat transfer devices incorporated into gloves or shoes could augment the use, or be used in lieu, of currently available cooling garments. The heat transfer devices used in this study used a pressure differential to maximize heat exchange. It has been demonstrated that, under certain conditions, substantial heat can be extracted from the body through the non-hairy skin surfaces without the application of a pressure differential [12-17]. The application of a cool load alone to the non-hairy skin surfaces may provide a benefit to heat sensitive individuals with MS under some heat-stress conditions.

## Conclusion

The results from this preliminary study demonstrate that, under a set of specific test conditions and in a small group of subjects who were selected in part because they engaged in regular exercise, utilization of the heat transfer capacity of the non-hairy skin surfaces can enable temperature-sensitive individuals with MS to extend participation in physical activities. Systematic longitudinal studies in larger cohorts of MS patients with specific deficits and levels of disability conducted under a variety of test conditions in independent research facilities are needed to confirm these preliminary findings. If these initial findings are replicated in future studies, the development of small wearable heat transfer equipment in the form of gloves or footwear could provide a benefit to individuals with MS.

## Competing interests

Patents have been issued for the technology disclosed in this manuscript [D. Grahn and H.C. Heller (Inventors); Stanford University (Assignee)], and Stanford University has entered into a licensing agreement with AVAcore Technologies, Inc., for the commercialization of the technology. Included in the license is a royalty agreement that grants Stanford University a percentage of the net sales of the technology, which will be shared by the University and the inventors. D. Grahn and H.C. Heller are founders of AVAcore Technologies but receive no ongoing compensation from the company. AVAcore Technologies provided no financial support for this research. To eliminate the possibility of this potential conflict of interest influencing the outcome of the research Stanford University requires that Grahn and Heller have no participation in the recruitment of subjects, the conduct of the experimental trials, and/or the analysis of the data.

## Authors' contributions

DAG, JvLSM, and HCH made substantial intellectual contributions to this study. DAG, JvLSM, and HCH were involved in the conception and design of the study, the interpretation of the data, and the drafting and revising of the manuscript. JvLSM was responsible for recruitment of the subjects, data acquisition, and data analysis. All authors have read and approved the final manuscript.

## Pre-publication history

The pre-publication history for this paper can be accessed here:



## References

[B1] Brown TR, Kraft GH (2005). Exercise and rehabilitation for individuals with multiple sclerosis. Phys Med Rehabil Clin N Am.

[B2] Romberg A, Virtanen A, Ruutiainen J (2005). Long-term exercise improves functional impairment but not quality of life in multiple sclerosis. J Neurol.

[B3] Romberg A, Virtanen A, Ruutiainen J, Aunola S, Karppi SL, Vaara M, Surakka J, Pohjolainen T, Seppanen A (2004). Effects of a 6-month exercise program on patients with multiple sclerosis: a randomized study. Neurology.

[B4] Syndulko KJM, Woldanski A, Baumhefner RW, Tourtellotte WW (1996). Effects of temperature in multiple sclerosis: A review of the literature. Neurorehabil Neural Repair.

[B5] White LJ, Dressendorfer RH (2004). Exercise and multiple sclerosis. Sports Med.

[B6] MacAllister WS, Krupp LB (2005). Multiple sclerosis-related fatigue. Phys Med Rehabil Clin N Am.

[B7] van der Berg M, Dawes H, Wade DT, Newman M, Burridge J, Izadi H, Sackley CM (2006). Treadmill training for individuals with multiple sclerosis: a pilot randomised trial. J Neurol Neurosurg Psychiatry.

[B8] White AT, Wilson TE, Davis SL, Petajan JH (2000). Effect of precooling on physical performance in multiple sclerosis. Mult Scler.

[B9] Bergersen TK (1993). A search for arteriovenous anastomoses in human skin using ultrasound Doppler. Acta Physiol Scand.

[B10] Rowell LB (1974). Human cardiovascular adjustments to exercise and thermal stress. Physiol Rev.

[B11] Greenfield A, Hamilton W, Dow P (1963). The circulation through the skin. Handbook of physiology Section 2: Circulation.

[B12] Allsopp AJ, Poole KA (1991). The effect of hand immersion on body temperature when wearing impermeable clothing. J R Nav Med Serv.

[B13] House JR, Holmes C, Allsopp AJ (1997). Prevention of heat strain by immersing the hands and forearms in water. J R Nav Med Serv.

[B14] Livingstone SD, Nolan RW, Cattroll SW (1989). Heat loss caused by immersing the hands in water. Aviat Space Environ Med.

[B15] Livingstone SD, Nolan RW, Keefe AA (1995). Heat loss caused by cooling the feet. Aviat Space Environ Med.

[B16] Selkirk GA, McLellan TM, Wong J (2004). Active versus passive cooling during work in warm environments while wearing firefighting protective clothing. J Occup Environ Hyg.

[B17] Tipton MJ, Allsopp A, Balmi PJ, House JR (1993). Hand immersion as a method of cooling and rewarming: a short review. J R Nav Med Serv.

[B18] Vanggaard L, Eyolfson D, Xu X, Weseen G, Giesbrecht GG (1999). Immersion of distal arms and legs in warm water (AVA rewarming) effectively rewarms mildly hypothermic humans. Aviat Space Environ Med.

[B19] Grahn D, Brock-Utne JG, Watenpaugh DE, Heller HC (1998). Recovery from mild hypothermia can be accelerated by mechanically distending blood vessels in the hand. J Appl Physiol.

[B20] Grahn DA, Cao VH, Heller HC (2005). Heat extraction through the palm of one hand improves aerobic exercise endurance in a hot environment. J Appl Physiol.

[B21] Hagobian TA, Jacobs KA, Kiratli BJ, Friedlander AL (2004). Foot cooling reduces exercise-induced hyperthermia in men with spinal cord injury. Med Sci Sports Exerc.

[B22] Hsu AR, Hagobian TA, Jacobs KA, Attallah H, Friedlander AL (2005). Effects of heat removal through the hand on metabolism and performance during cycling exercise in the heat. Can J Appl Physiol.

[B23] Craig AD, Bushnell MC (1994). The thermal grill illusion: unmasking the burn of cold pain. Science.

[B24] Guthrie TC (1950). Visual and motor changes in multiple sclerosis as a result of induced temperature changes. Trans Am Neurol Assoc.

[B25] Guthrie TC (1951). Visual and motor changes in patients with multiple sclerosis; a result of induced changes in environmental temperature. AMA Arch Neurol Psychiatry.

[B26] Guthrie TC, Nelson DA (1995). Influence of temperature changes on multiple sclerosis: critical review of mechanisms and research potential. J Neurol Sci.

[B27] Baker DG (2002). Multiple sclerosis and thermoregulatory dysfunction. J Appl Physiol.

[B28] Beenakker EA, Oparina TI, Hartgring A, Teelken A, Arutjunyan AV, De Keyser J (2001). Cooling garment treatment in MS: clinical improvement and decrease in leukocyte NO production. Neurology.

[B29] Schwid SR, Petrie MD, Murray R, Leitch J, Bowen J, Alquist A, Pelligrino R, Roberts A, Harper-Bennie J, Milan MD (2003). A randomized controlled study of the acute and chronic effects of cooling therapy for MS. Neurology.

[B30] Zhu M, Ackerman JJ, Sukstanskii AL, Yablonskiy DA (2006). How the body controls brain temperature: the temperature shielding effect of cerebral blood flow. J Appl Physiol.

[B31] Hayward JN, Baker MA (1968). Role of cerebral arterial blood in the regulation of brain temperature in the monkey. Am J Physiol.

[B32] Ereth MH, Lennon RL, Sessler DI (1992). Limited heat transfer between thermal compartments during rewarming in vasoconstricted patients. Aviat Space Environ Med.

[B33] Kurz A, Sessler DI, Birnbauer F, Illievich UM, Spiss CK (1995). Thermoregulatory vasoconstriction impairs active core cooling. Anesthesiology.

[B34] Ku YT, Montgomery LD, Lee HC, Luna B, Webbon BW (2000). Physiologic and functional responses of MS patients to body cooling. Am J Phys Med Rehabil.

[B35] Mourot L, Bouhaddi M, Gandelin E, Cappelle S, Dumoulin G, Wolf JP, Rouillon JD, Regnard J (2008). Cardiovascular autonomic control during short-term thermoneutral and cool head-out immersion. Aviat Space Environ Med.

